# Potential Value of a Combination of *Polypodium leucotomos* and *Aspalathus linearis* Extracts in Protecting Vitamin D Receptor Levels During Skin Oxidative Stress

**DOI:** 10.3390/ph19030494

**Published:** 2026-03-17

**Authors:** Marta Mascaraque, María Gallego-Rentero, Andrea Barahona-López, Paula Cano, Ángeles Juarranz, Ana López Sánchez, Salvador González

**Affiliations:** 1 Department of Biology, Universidad Autónoma de Madrid, 28049 Madrid, Spain; marta.mascaraque@uam.es (M.M.); maria.gallego@uam.es (M.G.-R.); andrea.barahona@uam.es (A.B.-L.); paula.villa2000@gmail.com (P.C.); 2 I+R+D Department, Cantabria Labs, 28760 Madrid, Spain; 3 Department of Medicine and Medical Specialties, University of Alcalá de Henares, 28805 Madrid, Spain; salvagonrod@gmail.com

**Keywords:** vitamin D receptor (VDR), oxidative stress, photoprotection, cancer prevention, *Aspalathus linearis*, *Polypodium leucotomos*

## Abstract

**Background/Objectives:** Vitamin D (VD), through the interaction with its receptor (VDR), plays essential roles in the skin. VDR-mediated signaling prevents cancer development and improves prognosis, making it an appealing target for therapy. However, VD cutaneous synthesis begins with solar exposure, which is the first etiological factor for cutaneous cancer and increases oxidative stress (OS). This complicates the dermatologist’s perspective when advising photoprotective strategies while aiming to consider the benefits of VD signaling. In this context, and in the absence of cutaneous data to date, this research aims to address VDR dynamics in skin cells and tissue subjected to OS. It also explores the potential of a natural photoprotectant with antioxidant properties (a specific combination of *Polypodium leucotomos* and *Aspalathus linearis* extracts) in preventing VDR depletion. **Methods:** HaCaT cell cultures and skin explants were used as experimental models. OS was induced by treatments with hydrogen peroxide (H_2_O_2_). The proteins of interest (VDR and Nuclear Factor Erythroid 2-Related Factor 2 (NRF2)) were analyzed by immunostaining. Cell viability, nuclear counterstaining, and Haematoxylin/Eosin staining were used as cyto/histochemical controls. **Results:** In both experimental models, we observed the reduction of VDR under OS. Pre-treatments with the botanical ingredient preserved VDR levels from that decline, probably through a mechanism involving NRF2. **Conclusions:** Cutaneous VDR levels are altered under oxidative stress, and certain photoprotectants could preserve them. This opens the door to preserving the benefits of VDR signaling while preventing solar radiation damage, bringing a new viewpoint for designing future strategies in skin cancer prevention and treatment.

## 1. Introduction

Vitamin D (VD) was first discovered as a liposoluble vitamin whose supplementation cured rickets, but its active form, 1,25 (OH)D_3_, is currently considered a hormone [1,2,3]. Its synthesis begins in the basal layer of the epidermis, where ultraviolet B (UVB) photons from sunlight convert 7-dehydrocholesterol into pre-D_3_ [4]. Then, an isomerization [5,6] and two subsequent hydroxylations are required to obtain the active form (1,25 (OH)D_3,_ calcitriol) [5]. Among its functions, VD facilitates the correct absorption of phosphorus and calcium, a process directly related to its role in rickets [7]. However, numerous biological processes in other tissues have been assigned to VD [1]. This is the case for its role in immune [8], cardiovascular [9], neurological [10], or cancerous processes [11]. For the main part of these functions, VD binds to its cellular receptor, VDR (vitamin D receptor) [6]. Reflecting the importance of VD in different tissues, VDR expression is not restricted to bone tissue but is also present in other cellular types, such as skin keratinocytes [12]. In fact, keratinocytes also express all the necessary enzymes to process D_3_ into the active form and trigger the intracellular signaling pathway through VDR [13]. Thus, today it is clear that the skin is not just the source tissue of vitamin D precursors but also one of its targets, affecting different aspects of skin physiology [5].

Due to its important roles in modulating the *stratum basale* proliferation and the subsequent differentiation of the keratinocytes, VDR has often been associated with skin cancer development [14]. Interestingly, controversial results have been found in studies trying to establish the relationship between VD serum levels and cutaneous cancer [15,16]. Intriguingly, in the case of non-melanoma skin cancer (NMSC), a positive correlation has been observed between VD serum levels and basal cell carcinoma (BCC) or squamous cell carcinoma (SCC) [17,18], probably due to higher sun radiation exposure in the NMSC groups [15,16,17,18]. However, this is currently a hot topic where a lack of rigorous studies with clear results prevents the main guidelines of skin cancer management from providing any recommendations to clinicians [15]. On the contrary, a robust amount of consistent evidence supports the role of VDR signaling in cutaneous cancer [19]. Mechanistically, VDR seems to be involved in DNA repair through its interaction with the nucleotide excision repair (NER) machinery [20]. Accordingly, VDR deficit slows down the DNA repair of cyclobutane pyrimidine dimers (CPDs) and pyrimidine(6,4)pyrimidone photoproducts (6–4PPs), which increases the mutation accumulation and the cancerous potential of solar radiation [21]. In addition, in the context of low VDR levels, the activation of Sonic the Hedgehog (SHH) and β-catenin pathways leads to a misregulation of the proliferation and differentiation of keratinocytes associated with cancer [14]. Moreover, some lncRNAs related to tumor development are over-induced in null VDR models [22]. These molecular pathways seem to underlie more clinically relevant observations. For example, VDR mutations or deletions predispose to cutaneous tumors [23,24,25]; deletions of both VDR and the calcium-sensing receptor are especially pro-oncogenic [19,26]; low VDR levels correlate with infiltrative basal cell carcinomas [27]; and VDR polymorphisms influence skin cancer risk [28]. Thus, beyond the controversial VD systemic status, ensuring skin VDR levels becomes an attractive target for the prevention and therapy of some skin cancers [29].

In this context, the relationship between oxidative stress (OS) and VDR should be considered. On one hand, several studies correlate higher levels of OS with lower serum levels of VD [30]. Additionally, adequate VD serum levels appear to help manage OS by reducing deleterious reactive oxygen species (ROS) [31], and some reports position OS at the centre of the VD paradox in cutaneous cancer [32]. On the other hand, some studies reveal the specific impact of OS on VDR levels [30]. In particular, Jain et al. demonstrated in 2018 how OS (indicated by low levels of reduced glutathione (GSH)) correlates with low levels of 25(OH)D_3_, VDR, and the endogenous antioxidant apparatus, including proteins such as NRF2 (Nuclear Factor Erythroid 2-Related Factor 2) [33]. They also observed how actively incrementing GSH induces higher VDR levels in different tissues [33]. These results point to OS as a key factor regulating VDR levels. However, no research has been performed addressing the impact of OS on VDR, specifically on the skin.

Considering the mentioned relevance of VD signaling in the skin and the importance of VDR levels for skin cancer, we aimed to study the effect of OS on VDR levels in skin cells and tissues. First, we established an experimental model in keratinocytes (HaCaT cells) to address VDR dynamics under OS induced by H_2_O_2_ treatments. Then, we evaluated the potential benefits of a botanical photoprotectant in the observed VDR dynamics. This plant-derived technology results from a mixture of two different *Aspalathus linearis* extracts (unfermented (ALU) and fermented (ALF)) with a wide spectrum of bioactivities [34,35] including chemopreventive potential [36,37] and light filtering activity [38] and a specific extract of *Polypodium leucotomos* (PLE) with comprehensively studied photoprotective properties [39]. Interestingly, recent works unveiled certain synergistic effects of that specific combination (PLE/ALU/ALF, commercialized as Aspa-Fernblock^®^) with antioxidant and photoprotective properties [40]. Finally, we reproduced the results observed in a whole tissue experimental model using skin explants.

In summary, this work aims to address clinicians’ concerns pertaining to VD signaling and skin cancer, where maintaining certain VDR levels appears to be crucial [5,19,29]. Importantly, it unveiled the possibility of using natural extracts with photoprotective and antioxidant capabilities (PLE/ALU/ALF) under this aim. The results presented herein are then particularly relevant because solar radiation exposure is involved in the first steps of VD production, but also induces direct DNA damage and generates substantial amounts of ROS, underscoring its role as the first etiological factor in skin cancer [41]. Then, by characterizing the reduction in VDR levels due to OS and the protective effect of a combination of plant extracts (PLE/ALU/ALF), this work supports the role of OS in regulating VDR levels and opens the doors to future recommendations for clinicians, focusing on preserving the benefits of VDR signaling while reducing the harmful effects of solar radiation, which could establish a new paradigm in skin cancer prevention and management.

## 2. Results

### 2.1. H_2_O_2_-Induced Oxidative Stress Reduces VDR in HaCaT Cells

**Figure 1:**
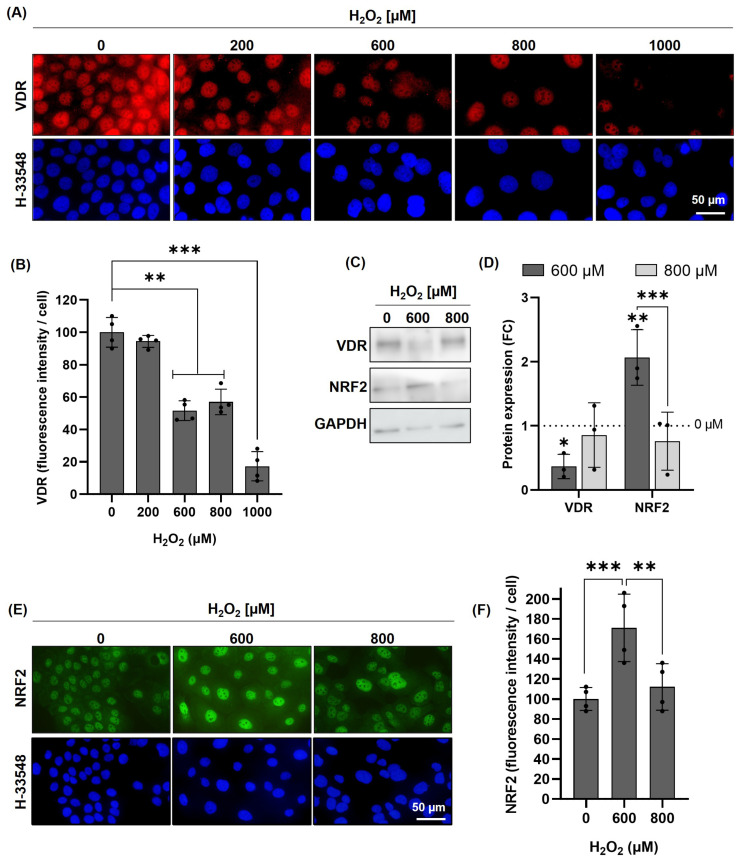
H_2_O_2_-induced oxidative stress reduces VDR in HaCaT cells. (**A**) Effect of OS on VDR levels using HaCaT cells after 24 h of H_2_O_2_ treatment at different concentrations. (**B**) Quantification of the VDR mean fluorescence intensity relative to the nucleus. (**C**) VDR and NRF2 protein levels by Western blot. Representative lanes of each protein, including GAPDH as a constitutive control. (**D**) VDR and NRF2 protein level quantification (Western blot) from three independent experiments. (**E**) Effect of OS on NRF2 expression in HaCaT cells after 24 h of H_2_O_2_ treatment at different concentrations. (**F**) Quantification of the NRF2 mean fluorescence intensity relative to the nucleus. In the immunofluorescence quantification analysis (**B**,**F**), for each replicate, at least 500 nuclei were analyzed. In all bar graphs, sample values are represented by overlapping dots, and the height of the bars represents the mean of each group ± SD (*: *p* < 0.05; **: *p* < 0.01; ***: *p* < 0.001; *n* ≥ 3).

The skin is one of the organs most affected by OS, primarily due to UV radiation. Given the evidence that VDR levels inversely correlate with systemic OS, we designed a model to address this specifically in skin cells (Figure S1A). We treated HaCaT cells with different doses (0–1000 µM) of hydrogen peroxide (H_2_O_2_). First, we analyzed the impact of H_2_O_2_ on cell survival, observing that doses between 600 and 800 µM induced a 30% lethality. Lower doses did not affect cell survival, while the highest dose (1000 µM) caused excessive damage, which may have distorted the following results (Figure S2A). Subsequently, the effect of those doses of H_2_O_2_ on VDR expression was evaluated using immunofluorescence. While the lowest dose (200 µM) did not induce relevant changes in VDR expression, doses of 600 µM and above led to a significant reduction (Figure 1A,B). Based on these results, doses of 600 and 800 µM were selected for further analyses, as they induced an evident reduction in VDR expression with a mild impact on cell lethality. Figure 1C (full membrane Figure S1B) and Figure 1D show the protein quantification by Western blot, confirming a significant decrease in VDR levels at 600 µM of H_2_O_2_.

NRF2 is a key regulator of antioxidant enzyme expression [42]. Therefore, we assessed whether our selected doses of H_2_O_2_ would induce NRF2. To investigate this, we evaluated NRF2 in HaCaT cells treated with selected doses of H_2_O_2_ using Western blot (Figure 1C,D) and immunofluorescence (Figure 1E,F), which showed a significant increase in NRF2 at 600 µM H_2_O_2_. These results indicate that at this concentration, cells detect oxidative stress and activate their antioxidant defence response. In contrast, at 800 µM H_2_O_2_, the increase in NRF2 was not statistically significant, suggesting a possible impairment in the cellular response at higher oxidative stress levels.

This is the first evidence that oxidative stress tends to reduce VDR levels in skin cells and suggests a dose-dependent interplay between VDR levels, oxidative damage, and cellular antioxidant responses.

### 2.2. Treatments with PLE/ALU/ALF Prevent VDR Repression Induced by Oxidative Stress in HaCaT Cells

**Figure 2:**
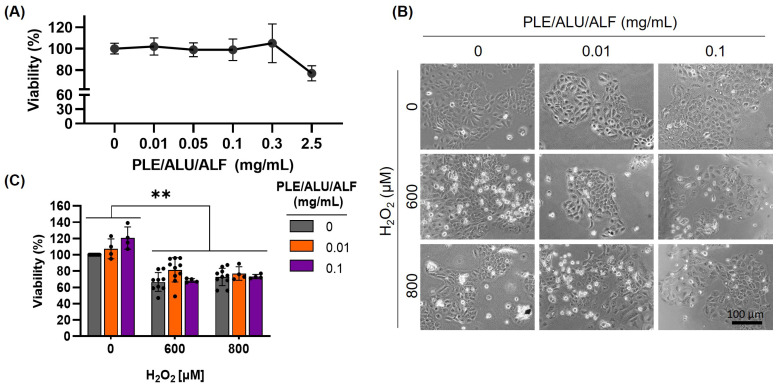
PLE/ALU/ALF’s effect on cell viability. (**A**) Cell viability effect after 0–2.5 mg/mL of PLE/ALU/ALF analyzed by MTT. Values were represented as the mean of each group ± SD (*n* ≥ 3). (**B**) Cell morphology. (**C**) Cell viability assay under OS induced by H_2_O_2_ (600 and 800 µM) after pre-treatments with PLE/ALU/ALF (0.01 and 0.1 mg/mL). Sample values are represented by overlapping dots, and the height of the bars represents the mean of each group ± SD (**: *p* < 0.01; *n* ≥ 3).

After establishing a suitable experimental model to study VDR dynamics under OS, the photoprotective technology PLE/ALU/ALF was evaluated. First, a preliminary toxicity analysis (cell survival assay) was performed to determine the PLE/ALU/ALF concentrations (0–2.5 mg/mL) that do not severely impact the experimental model. Considering the absence of a cytotoxic effect until the highest dose (2.5 mg/mL, Figure 2A), doses of 0.01 and 0.1 mg/mL were selected for subsequent experiments. Pre-treatments with PLE/ALU/ALF did not significantly modify the cellular viability reduction induced by H_2_O_2_ (Figure 2B,C).

**Figure 3:**
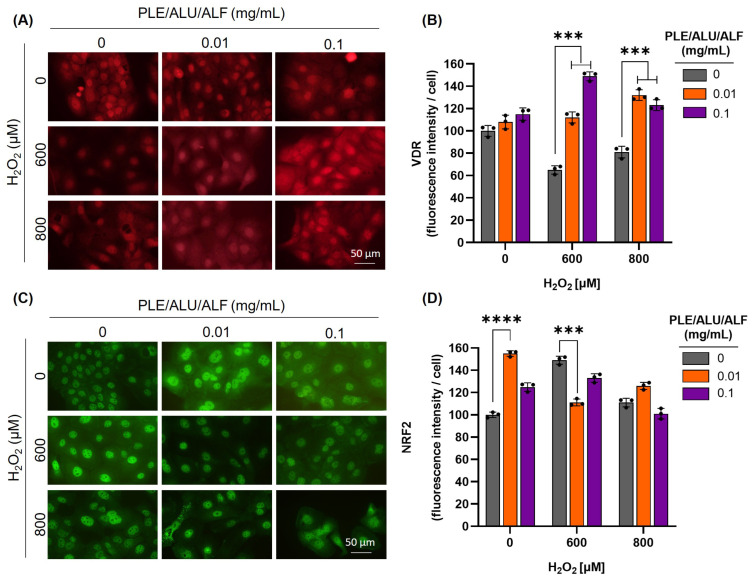
PLE/ALU/ALF prevents VDR depletion induced by oxidative stress. Effect of PLE/ALU/ALF (0.01 and 0.1 mg/mL), OS induced by H_2_O_2_ (600 and 800 µM), and the combination of both treatments on VDR and NRF2 protein levels. (**A**) Representative microscopy photographs of the VDR immunofluorescence. (**B**) VDR mean fluorescence intensity relative to the nucleus. (**C**) Representative microscopy photographs of NRF2 immunofluorescence. (**D**) NRF2 mean fluorescence intensity relative to the nucleus (Höechst staining shown in Figure S3). In the immunofluorescence quantification (**B**,**D**), for each replicate, at least 500 nuclei were analyzed, and the height of the bars represents the mean of each group ± SD (***: *p* < 0.001; ****: *p* < 0.0001; *n* = 3).

We then evaluated the effect of PLE/ALU/ALF on VDR protein levels using immunofluorescence. In the absence of OS, no significant differences were observed with any of the PLE/ALU/ALF doses tested compared to the control. However, the treatments with the botanical photoprotectant of interest were able to prevent the VDR reduction induced by OS (Figure 3A,B and Figure S3). To further investigate the molecular basis of the observed phenotype, we analyzed NRF2 protein levels. In the absence of OS, treatments with PLE/ALU/ALF (0.01 mg/mL) induced NRF2 expression, suggesting a certain activation of the endogenous antioxidant pathway. Interestingly, under OS conditions, pre-treatments with PLE/ALU/ALF maintained NRF2 comparable to basal levels, indicating a potential protective effect against H_2_O_2_-induced oxidative damage (Figure 3C,D and Figure S3). These results highlight the effect of PLE/ALU/ALF on NRF2 modulation, as well as its protective potential in maintaining VDR levels under OS conditions.

### 2.3. PLE/ALU/ALF Treatments Prevent VDR Repression Induced by Oxidative Stress in Ex Vivo Models

Based on the promising results obtained in the HaCaT cell line, we proceeded to evaluate VDR dynamics and the effect of PLE/ALU/ALF in an ex vivo model. For this purpose, ex vivo skin samples from a 60-year-old female donor were pre-treated with PLE/ALU/ALF for 24 h (0.01 and 0.1 mg/mL) prior to H_2_O_2_ administration (1.25, 2.5, and 7 mM, 2 h) (Figure S1B).

**Figure 4:**
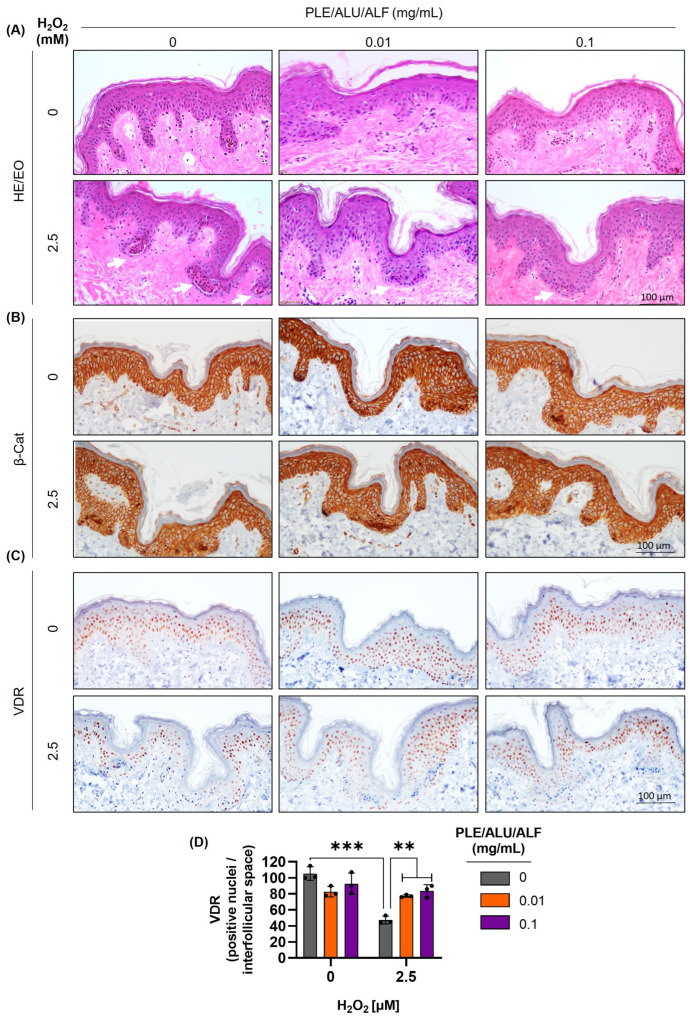
PLE/ALU/ALF prevents VDR depletion induced by oxidative stress in ex vivo models. Effect of PLE/ALU/ALF (0.01 and 0.1 mg/mL), H_2_O_2_ (2.5 mM), and the combination of both treatments in ex vivo skin samples. (**A**) HE/EO staining (hematoxylin and eosin stain). White arrows: necrotic areas. (**B**) β-catenin immunohistochemistry. (**C**) VDR immunohistochemistry. (**D**) VDR quantification as the positive nuclei/interfollicular space. Sample values are represented by overlapping dots, and the height of the bar represents the mean of each group ± SD (**: *p* < 0.01; ***: *p* < 0.001; *n* = 3).

First, we evaluated tissue integrity by hematoxylin and eosin staining. With the three doses of H_2_O_2_ evaluated, an increment in the number of necrotic areas (indicated by white arrows) was observed (intermediate concentration—2.5 mM—shown in Figure 4A; entire panel in Figure S4). Interestingly, pre-treatments with PLE/ALU/ALF reduced the occurrence of these necrotic areas. Additionally, PLE/ALU/ALF treatments did not induce any morphological changes in either the epidermis or dermis (Figure 4A and Figure S4).

Subsequently, we verified that the epidermal structure was preserved with both doses of PLE/ALU/ALF by immunohistochemistry of the adhesion protein β-catenin. Hematoxylin and eosin results were reproduced, showing that the β-catenin staining had a lower number of H_2_O_2_-induced dead areas under PLE/ALU/ALF pre-treatments (Figure 4B and Figure S5).

Finally, the VDR protein was evaluated and quantified. As observed in HaCaT cells, PLE/ALU/ALF pre-treatments did not induce significant changes compared to the control condition. Also, in accordance with the results obtained in the cellular model, the OS induced by H_2_O_2_ treatments significantly reduced the number of VDR-positive nuclei per interfollicular space, and PLE/ALU/ALF was able to prevent this effect (Figure 4C,D and Figure S6).

## 3. Discussion

There is a general concern about serum VD levels and the perception that sun exposure confers related health benefits, which is impacting the preventive measures against skin cancer. However, the current literature presents conflicting findings [15,16]. While some studies suggest no clear association between serum vitamin D concentrations and skin cancer prevention or prognosis, others highlight a coherent role of VD signaling through its receptor, VDR [5,19,29]. Thus, dermatologists face a complex decision when advising patients, as UVB radiation is essential for VD synthesis [4]; but it remains the primary etiological factor for skin cancer [41]. On a complementary note, OS impacts both VD and VDR systemic levels [30,32,33], but there are no records of its skin’s dynamics. Thus, aiming to shed light on this field and address clinicians’ concerns, we investigated VDR levels under OS conditions in both cultured keratinocytes and skin tissue, and explored the effect of the photoprotective ingredient PLE/ALU/ALF.

This study presents, for the first time, how OS reduces VDR levels in skin cells and tissue. We used specifically H_2_O_2_ as a stressor instead of sun radiation, aiming to assess the impact of OS on VDR levels independently from VD variations (as VD synthesis is activated by UVB exposure [4]). This separation is particularly relevant in the context of skin photoprotection, because unprotected sun exposure would potentially increase oxidative stress but also VD levels. Considering that VD has been reported to stimulate VDR expression [43,44], radiation-induced VD levels could consequently increase VDR expression, masking the results obtained and complicating their interpretation. Therefore, differentiating between the effects of OS induced by H_2_O_2_ and sun radiation allows for a better understanding of their distinct roles in VDR regulation, and opens doors to their application within photoprotective mechanisms.

VDR plays essential roles in maintaining skin integrity and preventing the development of skin cancer [45,46]. For instance, silencing VDR has been shown to trigger keratinocyte hyperproliferation and increased cyclin D1 expression, while VD/VDR also influences β-catenin/TCF signaling. Moreover, the absence of VDR enhances susceptibility to UVB-induced carcinogenesis, highlighting its role in preventing skin cancer [47]. Our results demonstrate that H_2_O_2_ reduces VDR levels, which could facilitate the onset of skin cancer. Thus, in the context of photoprotection strategies, attention should be given not only to maintaining adequate VD levels but also to preserving VDR expression. Given that the VD signaling involved in skin cancer prevention is VDR-dependent, sun exposure would always be counterproductive, because sun radiation causes direct DNA damage [48], and even if it could increase VD synthesis, it would tend to reduce VDR levels due to the OS generated.

In this context, PLE/ALU/ALF results appear to be of interest, as it would help maintain VDR levels under OS conditions while being a photoprotective technology. This combination of plant extracts (commercialized as Aspa-Fernblock^®^) includes a specific extract of *Polypodium leucotomos* and a mixture of two different extracts of *Aspalathus linearis* standardized in a particular chemical profile [40]. Both types of natural extracts are supported by numerous reports unveiling their photoprotective character [38,39,40], a bioactivity that at least partially emerges from their capacity to stimulate the cell’s endogenous mechanisms against UV-induced damage [40,49,50]. Among those mechanisms, NRF2 is considered a master regulator that transcriptionally modulates the endogenous antioxidant machinery [42,51,52]. As a relevant part of the UV-associated deleterious effects involves an increment in OS, NRF2 is also key in regulating the skin’s endogenous responses against UV [53,54], emerging as a promising pharmacological target in the field of photoprotection [55,56]. Furthermore, NRF2 has been reported to underlie the beneficial effects of *Aspalathus linearis* [57,58] and *Polypodium leucotomos* [59,60]. In our cellular model, we observed NRF2 induction in response to H_2_O_2_ treatments, reflecting the cell’s perception of OS. Moreover, NRF2 levels inversely correlated with VDR levels, supporting the reduction in VDR levels in response to a cellular perception of OS. However, PLE/ALU/ALF pre-treatments appeared to preserve VDR levels despite oxidative challenge, suggesting that this botanical technology may prime the cell’s endogenous antioxidant defences. This could be facilitated by NRF2 expression or activation, fostering cellular resilience against oxidative stress. Thus, the results presented here would suggest that the PLE/ALU/ALF effect on VDR levels under OS may be NRF2-mediated. Nonetheless, as the current study is merely correlative, exploring the mechanistic aspects of VDR signaling in relation to OS and skin cancer would be interesting for the following research. In this scenario, there are previous reports supporting NRF2 regulation mediated by VDR in other tissues [61,62]. In fact, using SCC25 cells (from oral squamous carcinoma cells whose proliferation is arrested by vitamin D), VDR was found to bind the *NRF2* promoter region, suggesting *NRF2* gene expression is a direct target of VDR with potential benefits in the context of squamous cell carcinoma [63]. Intriguingly, our results show a certain induction of NRF2 while repressing VDR levels, which suggests a different regulatory mechanism. Considering other studies have found a correlation of VDR levels with genes such as *GCLC* and *GCLM* [33] (NRF2 transcriptional targets [64,65]), the relationship between VDR and NRF2 warrants further investigation.

This study should be considered as a first step in unravelling the bases of VDR dynamics, but undoubtedly, further experimental approaches should be performed, building on the current study’s limitations. One of the limitations to be addressed is the use of H_2_O_2_ as the only OS inducer. Considering the positive results reported, future research should include other OS inducers such as tert-butyl hydroperoxide (TBHP), as well as different wavelengths within the solar spectrum or full sun radiation. In addition, even when two experimental models were employed, both have important specificities that need to be taken into consideration. For example, employing other cellular models and/or ex vivo tissue from donors of different ages and phototypes would importantly help broaden the translational relevance of the findings. Likewise, the current work focused on VDR protein levels, but assessing functional analysis or dissecting VDR intracellular signaling would better define the scope and significance of the results.

Finally, translating these findings into clinical practice represents another vital avenue for future work. This could be important in vulnerable populations for whom the benefits of VD and VDR signaling have already been reported, such as vitiligo patients [66], but especially relevant for groups at a higher risk of epidermal cancer development, such as those with actinic keratosis [28,67], and Xeroderma Pigmentosum [20]. In cases where sun exposure should be strongly prevented, ingredients such as PLE/ALU/ALF, or formulations combining PLE/ALU/ALF with vitamin D or analogues could maximize sun benefits while minimizing the risks, justifying their clinical research.

Hence, this work investigates the influence of environmental factors on VDR regulation and opens doors to split sun exposure risks from its benefits. Our results support that these concepts could be implemented using natural extracts, preserving VDR benefits while reducing solar radiation harms. This study contributes to addressing current clinical concerns and could help establish a new paradigm for the design of future skin cancer prevention and management strategies, considering the inclusion of natural extracts.

## 4. Materials and Methods

### 4.1. Cell Types

For the in vitro studies, we utilized HaCaT cells, a spontaneously transformed but non-tumorigenic human keratinocyte cell line (Cell Line Service, Eppelheim, Germany). Cells were cultured in Dulbecco’s Modified Eagle’s Medium (DMEM) enriched with 10% (*v*/*v*) fetal bovine serum (FBS) and 1% antibiotics (penicillin, 100 units/mL; streptomycin 100 mg/mL) from Thermo Fisher Scientific Inc. (Rockford, IL, USA). Cultures were maintained under standard conditions of 5% CO_2_, 95% humidity, and 37 °C.

### 4.2. Ex Vivo Skin Samples

Ex vivo skin samples were obtained from Conda Labs (Madrid, Spain). Full-thickness skin discs (8 mm diameter), free of adipose tissue, from a 60-year-old female donor were used. Upon reception, skin explants were placed in culture medium (Conda Labs, Spain) and allowed to equilibrate for 48 h prior to treatment. All handling and subsequent experimental procedures were performed maintaining the explants under air–liquid interface conditions within the Petri dishes, ensuring tissue viability and preservation of the epidermal barrier.

### 4.3. Treatments

The botanical ingredient PLE/ALU/ALF is commercialized as Aspa-Fernblock^®^ and was supplied by Industrial Farmaceutica Cantabria, S.A. (Cantabria Labs, Madrid, Spain), which provided a stock solution of 10 mg/mL. For the stock solution preparation, the proper amount of dried powder was weighed (including the extracts in proportions of 50% PLE and 50% ALU/ALF, as tested in Caceres Estevez et al. 2025 [40]) and dissolved in water by stirring at 37 °C for 30 min, followed by filtering through a PVDP 0.45 μm membrane to remove solid residues and biological contaminants.

Treatments in HaCaT cells (Figure S1A) were performed when the cultures reached 30–40% confluence, using concentrations of 0.01 and 0.1 mg/mL (prepared from the 10 mg/mL stock) in DMEM with 10% FBS. After 24 h, hydrogen peroxide (H_2_O_2_) (0–1000 µM) in serum-free DMEM was added for another 24 h. Afterwards, the medium was switched back to complete media (DMEM + 10% FBS), and cells were further incubated for 24 h prior to evaluation. For ex vivo skin samples (Figure S1B), PLE/ALU/ALF (0.01 and 0.1 mg/mL) was added to fresh skin culture medium for 24 h. H_2_O_2_ (0–7 mM) was then added in PBS (phosphate-buffered saline) for 2 h, followed by a medium change 16 h before the evaluation by immunohistochemistry (IHC).

### 4.4. Cell Viability Assay

Cell viability was quantified via the MTT [3-(4,5-dimethylthiazol-2-yl)-2,5-diphenyltetrazolium bromide] assay (Sigma–Aldrich, St. Louis, MO, USA). Cultures received MTT at a final concentration of 100 μg/mL and were maintained at 37 °C for 3 h. Resulting formazan crystals were solubilized in dimethyl sulfoxide (DMSO), and absorbance was recorded at 542 nm with a SpectraFluor microplate reader (Tecan, Bradenton, FL, USA). Cytotoxicity was determined by expressing the proportion of surviving cells relative to untreated control cultures.

### 4.5. Immunostaining

Cells grown on coverslips were fixed in formaldehyde (3.7%) in PBS for 30 min, washed with PBS, and permeabilized for 30 min (0.1% Triton X-100-PBS). For immunostaining, cells were incubated with primary antibodies (VDR -Cell Signaling Technology Inc., Danvers, MA, USA-; NRF2 -Abcam, Cambridge, UK-) for 1 h at 37 °C in a humid chamber. After PBS washes, cells were incubated with the secondary antibodies AF546 goat anti-rabbit IgG or AF488 goat anti-mouse IgG (Thermo Fisher Scientific Inc.) for 45 min at 37 °C, washed with PBS, counterstained with Hoechst-33258 (1 µg/mL, Sigma-Aldrich, St. Louis, MO, USA), and mounted with ProLong^®^ (Life Technologies, Carlsbad, CA, USA). Images were captured using an Olympus BX-61 fluorescent microscope. For the immunofluorescence quantification, first, for each sample of each of the independent experiments, the protein signal of more than 500 nuclei relative to the number of corresponding nuclei (observed by Hoechst-33258 staining) was obtained. Then, the variables (mean, standard deviations, and statistical analysis) were calculated based on the data from at least 3 independent experiments. Then *n* ≥ 3 represents the data from at least 3 biological replicates.

### 4.6. Western Blots

Cell lysates were prepared in RIPA (radioimmunoprecipitation assay) buffer supplemented with Triton (pH 7.4; Bio-world, Dublin, OH, USA), phosphatase inhibitors (PhosSTOP EASYpack; Roche, Mannheim, Germany), and protease inhibitors (Complete ULTRA Mini EDTA-free EASYpack tablets; Roche, Basel, Switzerland), following the supplier’s guidelines. Total protein content was quantified using the BCA Protein Assay Kit (Thermo Scientific Pierce, Rockford, IL, USA). Extracts (30 µg protein/lane) were diluted in Laemmli buffer (Bio-Rad, Hercules, CA, USA) and heated at 98 °C for 5 min. Proteins were separated by SDS–PAGE on acrylamide/bisacrylamide gels and transferred to polyvinylidene difluoride (PVDF) membranes (Bio-Rad) using the Transblot Turbo transfer system (Bio-Rad). Membranes were blocked with skimmed milk in 0.1% TBS-Tween 20 and incubated with antibodies against VDR (Cell Signaling Technology, Inc., Danvers, MA, USA), NRF2 (MedChemExpress, Monmouth Junction, NJ, USA) and GAPDH (Abcam, Cambridge, UK), followed by peroxidase-conjugated secondary antibodies (Thermo Fisher, Rockford, IL, USA). Protein bands were visualized using the ECL Plus Kit (Amersham, Little Chalfont, UK) and the high-resolution ChemiDocTR XRS+ system (Bio-Rad). Images were digitized with Image Lab version 3.0.1 software (Bio-Rad).

### 4.7. Haematoxylin and Eosin Stain and Immunohistochemistry Analysis

Skin samples were formalin-fixed and paraffin-embedded (FFPE, Panreac, Barcelona, Spain) for histological and immunohistochemical staining. Tissue sections were deparaffinized and hydrated (10 min xylene; 5 min 100% alcohol; 5 min 96% alcohol; 5 min 70% alcohol), stained with haematoxylin and eosin (2 min with each stain) (Thermo Scientific Pierce, Rockford, IL, USA) and dehydrated following the reverse order of alcohols. Finally, they were mounted in DePeX (Serva, Heidelberg, Germany). For immunohistochemistry (IHC), endogenous peroxidase was blocked with 3% hydrogen peroxide (Panreac, Barcelona, Spain) in methanol. Antigen retrieval was conducted by immersing sections in citrate buffer (pH 6) in a pressure cooker for 10 min. After cooling, blocking was performed with non-immune serum (Dako, Agilent Technologies, Santa Clara, CA, USA) for 1 h at room temperature, followed by overnight incubation at 4 °C with primary antibodies (β-catenin -BD Biosciences, San Jose, CA, USA- and VDR -Cell Signaling Technology, Inc., Danvers, MA, USA-). Sections were then incubated with streptavidin-peroxidase-conjugated secondary antibodies (Cell Signaling Technology, Inc., Danvers, MA, USA) for 30 min at room temperature. Colour development was achieved using 3,3’-diaminobenzidine solution (DAB, Vector Laboratories, Burlingame, CA, USA) and counterstained with hematoxylin. Sections were dehydrated in graded alcohol series and mounted with DePeX (Serva, Heidelberg, Germany).

### 4.8. Optical Microscopy

Microscopy imaging employed an Olympus BX-61 epifluorescence microscope coupled to a DP70 CCD camera (Olympus, Tokyo, Japan), with UV (360–370 nm, UG-1 filter), blue (450–490 nm, UG-1 filter), or green (570–590 nm, DM 590 filter) excitation light filters and bright field. Photoshop CS5 (Adobe Systems Inc., San Jose, CA, USA) was utilized for fluorescence image processing.

### 4.9. Statistical Analysis

Results were presented as the mean ± standard deviation (SD). In each experiment, *n* ≥ 3. All experiments were replicated with similar results. GraphPad Prism version 9.0 (GraphPad Software Inc., Boston, MA, USA) was used for statistical analysis and graphical representation. Differences were assessed using analysis of variance (ANOVA, one-way) and post hoc Bonferroni’s test (*p* < 0.05). Significant differences were denoted as *: *p* < 0.05; **: *p* < 0.01; ***: *p* < 0.001; and ****: *p* < 0.0001.

## 5. Patents

The ingredient presented here (PLE/ALU/ALF, commercialized as Aspa-Fernblock^®^) is currently patent pending by Cantabria Labs.

## Data Availability

The original contributions presented in this study are included in the article and Supplementary Material. Further inquiries can be directed to the corresponding author.

## Supplementary Materials

The following supporting information can be downloaded at: https://www.mdpi.com/article/10.3390/ph19030494/s1, Figure S1: Schematic diagram of the time course treatment; Figure S2: Effect of oxidative stress on HaCaT cells; Figure S3: PLE/ALU/ALF protects from oxidative stress-induced VDR repression; Figure S4. Hematoxylin and eosin staining in ex vivo models; Figure S5. β-catenin immunohistochemistry in ex vivo models; Figure S6. VDR immunohistochemistry in ex vivo models.

## Abbreviations

The following abbreviations are used in this manuscript:

ALF	*Aspalathus linearis* fermented extract
ALU	*Aspalathus linearis* unfermented extract
BCC	Basal cell carcinoma
CPDs	Cyclobutane pyrimidine dimers
DAB	3,3’-diaminobenzidine solution
DMEM	Dulbecco’s Modified Eagle’s Medium
DMSO	Dimethyl sulfoxide
PLE	*Polypodium leucotomos* extract
FBS	Fetal bovine serum
FFPE	Formalin-fixed, paraffin-embedded
GSH	Reduced glutathione
H_2_O_2_	Hydrogen peroxide
IHC	Immunohistochemistry
MTT	3-(4,5-dimethylthiazol-2-yl)-2,5-diphenyltetrazolium bromide
NER	Nucleotide excision repair
NMSC	Non-melanoma skin cancer
NRF2	Nuclear Factor Erythroid 2-Related Factor 2
OS	Oxidative stress
PBS	Phosphate-buffered saline
PVDF	Polyvinylidene difluoride
6–4PPs	Pyrimidine(6,4)pyrimidone photoproducts
RIPA	Radioimmunoprecipitation assay buffer
ROS	Reactive oxygen species
SCC	Squamous cell carcinoma
SD	Standard deviation
SHH	Sonic the Hedgehog
UVB	Ultraviolet B
VD	Vitamin D
VDR	Vitamin D receptor
