# Supplementary material for: Potential Value of a Combination of Polypodium leucotomos and Aspalathus linearis Extracts in Protecting Vitamin D Receptor Levels During Skin Oxidative Stress
Source: Pharmaceuticals (Basel). 2026 Mar 17;19(3):494. doi: 10.3390/ph19030494 (PMC13029066; doi:10.3390/ph19030494)
Supplement: Supplementary file 1 [file pharmaceuticals-19-00494-s001.zip › pharmaceuticals-4181471-supplementary.pdf]

SUPORTING INFORMATION

Supporting figures

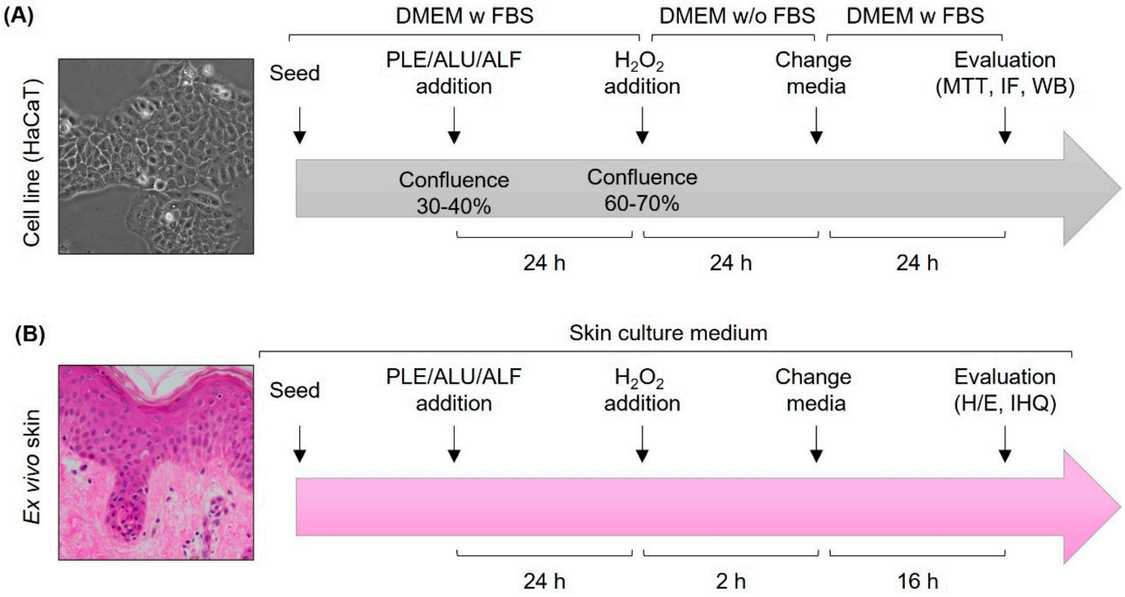

Figure S1. Schematic diagram of the time course treatment. (A) HaCaT cell line and **(B)** *ex vivo* skin.

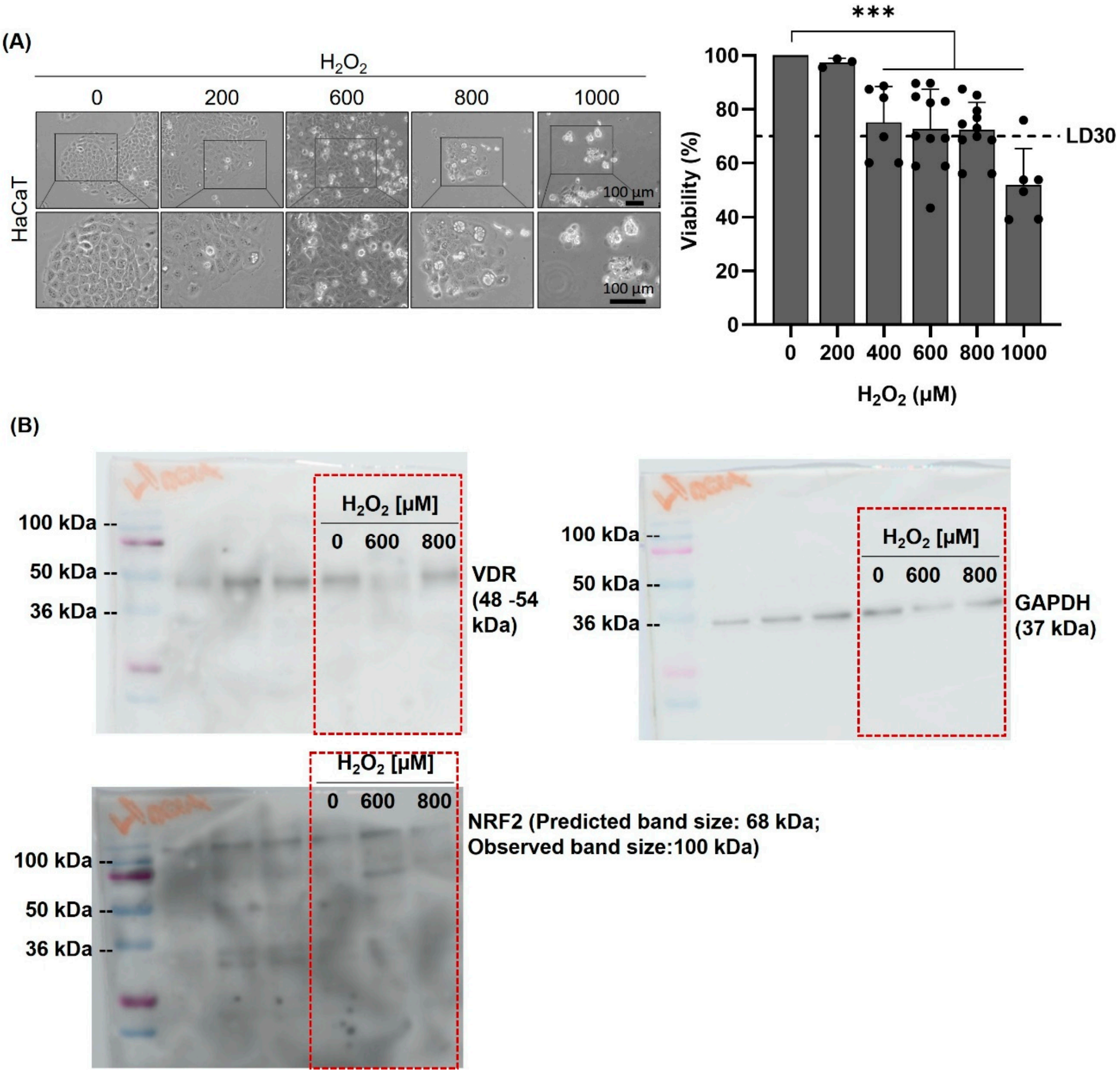

Figure S2. Effect of oxidative stress on HaCaT cells. A) Cell morphology and viability assay (relative to untreated cells) under OS induced by H_2O_2 (0-1000 μM). In the bar graph, sample values are represented by overlapping dots, and the height of the bars represents the mean of each group ± SD (∗∗∗: $p< 0.001$; $n \geq 3$). **B)** VDR, NRF2, and GAPDH protein levels by western blot. Original membrane for the representation of Figure 1C.

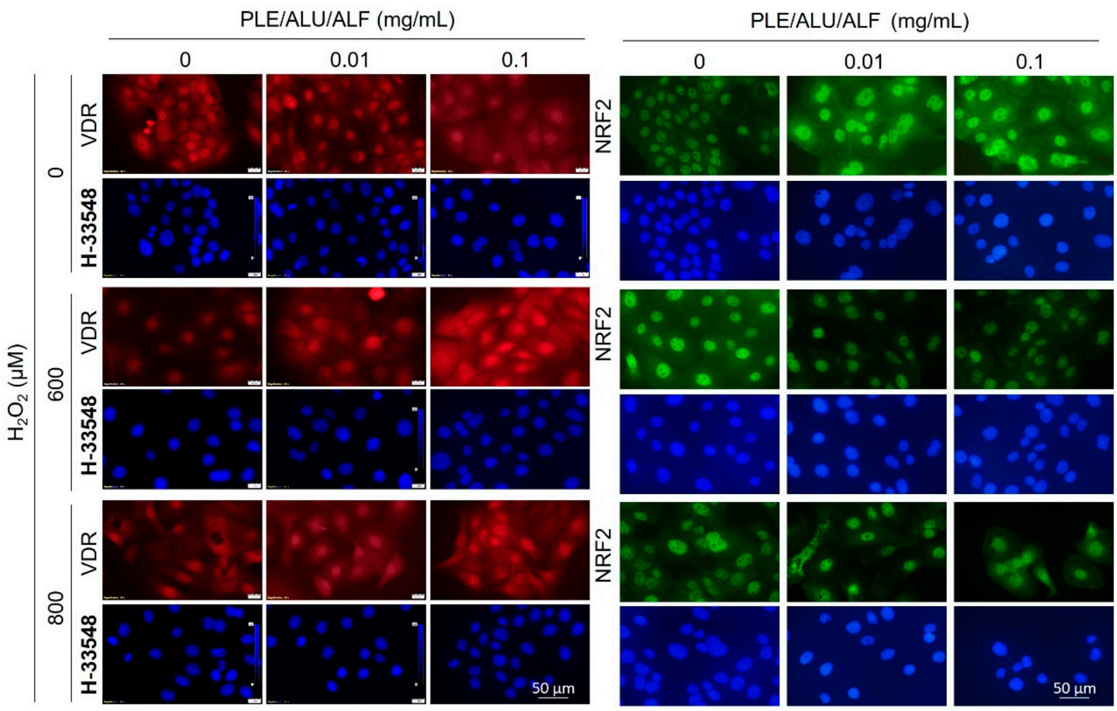

Figure S3. PLE/ALU/ALF protects from oxidative stress-induced VDR repression. Effect of PLE/ALU/ALF (0.01 and 0.1 mg/ml), H_2O_2 (600 and 800 µM), and their combination on VDR and NRF2 expression. Full immunofluorescence panel corresponding to Figure 3, including Höechst nuclear counterstaining.

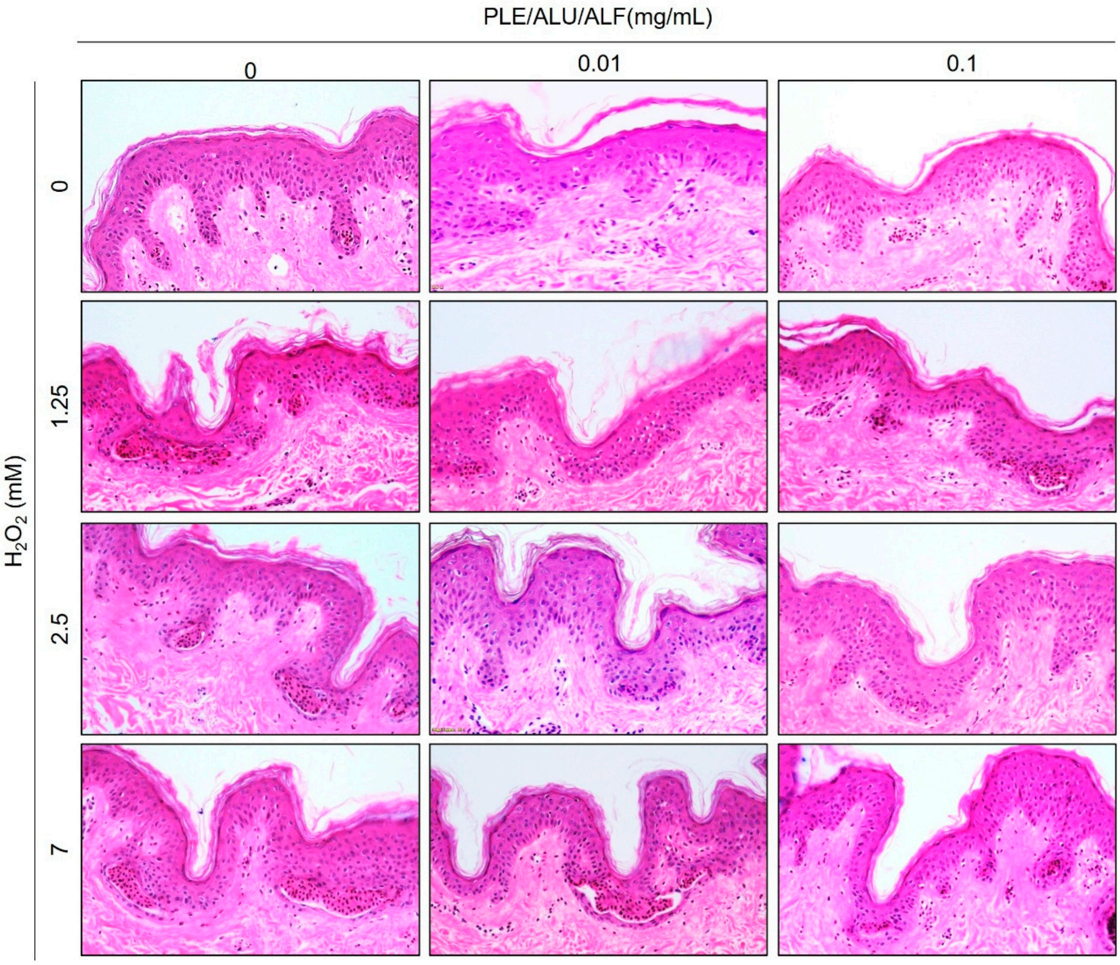

Figure S4. **Hematoxylin and eosin staining in *ex vivo* models**. Effect of PLE/ALU/ALF (0.01 and 0.1 mg/ml), H_2O_2 (1.25, 2.5, and 7 mM), and their combination on *ex vivo* skin samples.

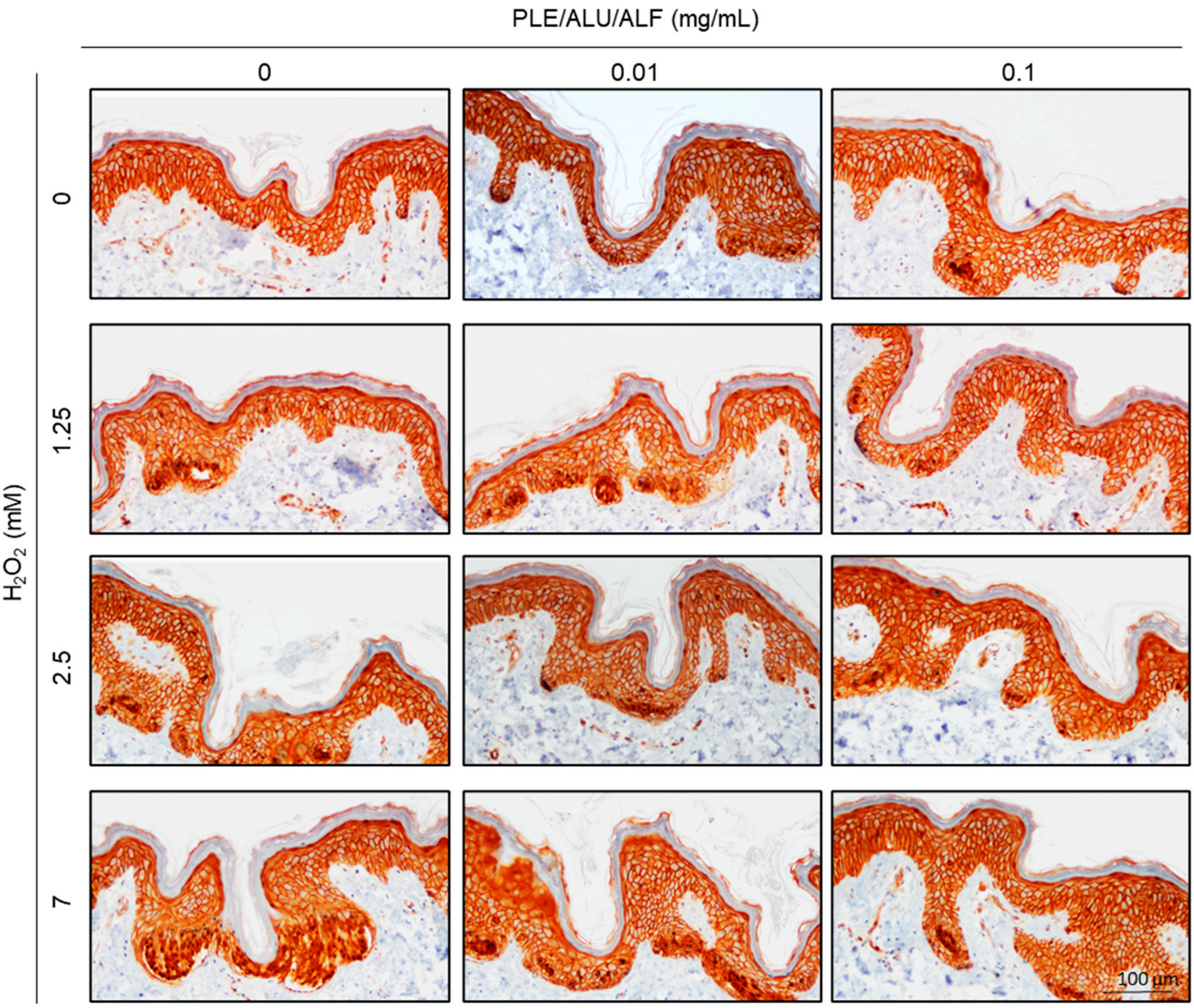

Figure S5. β-catenin immunohistochemistry in *ex vivo* models. Effect of PLE/ALU/ALF (0.01 and 0.1 mg/ml), H_2O_2 (1.25, 2.5, and 7 mM), and their combination on *ex vivo* skin samples.

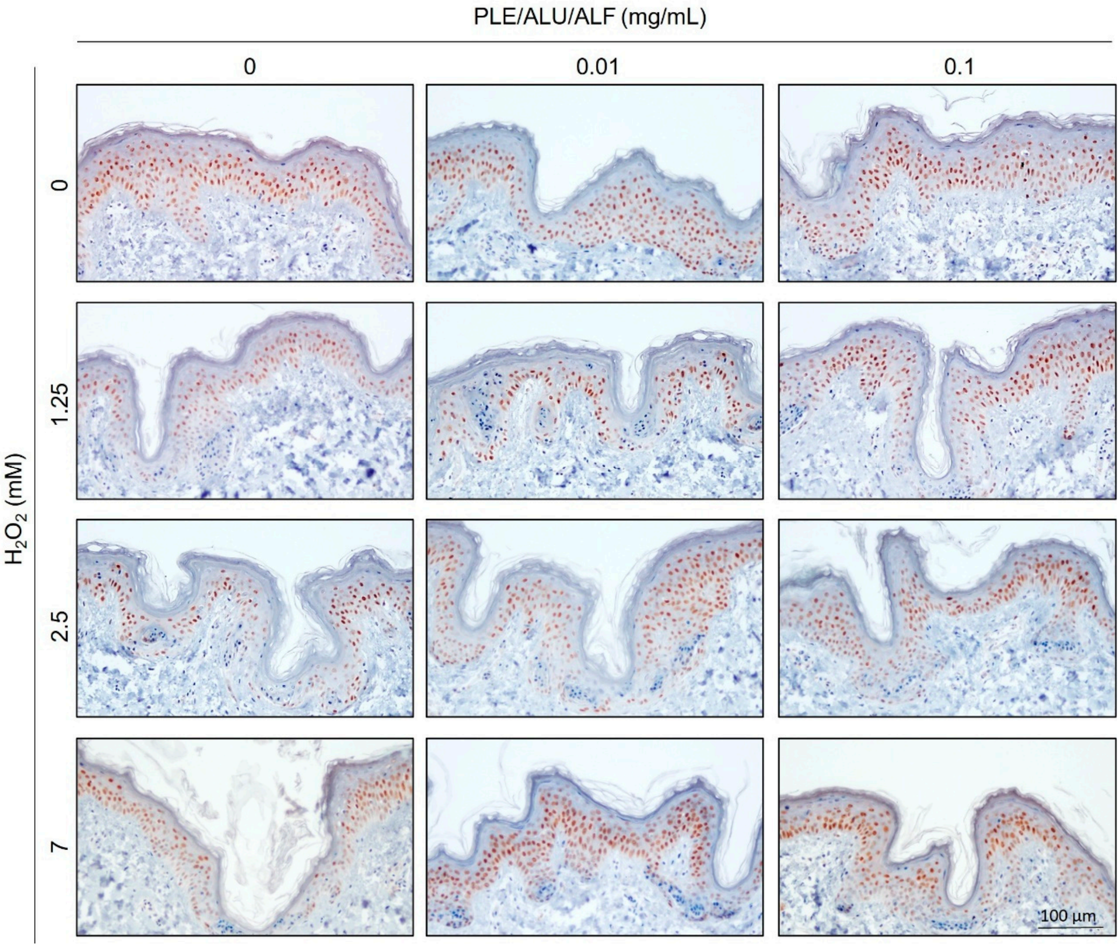

Figure S6. VDR immunohistochemistry in *ex vivo* models. Effect of PLE/ALU/ALF (0.01 and 0.1 mg/ml), H_2O_2 (1.25, 2.5, and 7 mM), and their combination on *ex vivo* skin samples.
